# Spontaneously perforated pyometra: an unusual cause of acute abdomen and pneumoperitoneum

**DOI:** 10.1308/003588412X13373405387410

**Published:** 2012-05

**Authors:** IM Shapey, T Nasser, P Dickens, M Haldar, MH Solkar

**Affiliations:** Tameside Hospital NHS Foundation Trust,UK

**Keywords:** Pyometra, Spontaneous perforation, Sepsis, Acute abdomen

## Abstract

Pneumoperitoneum is usually associated with gastrointestinal perforation or following surgical and endoscopic procedures. We report a rare case of spontaneously perforated pyometra presenting with generalised peritonitis and pneumoperitoneum. Perforation of the uterus is also unusual and often associated with the presence of an intrauterine device, a gravid uterus or malignancy. Our case illustrates the importance of clinical knowledge of acute and neoplastic gynaecological diseases, which are not uncommonly encountered by the general surgeon. Moreover, good appreciation of pelvic anatomy and close collaboration with gynaecology colleagues is essential as operative intervention is often required.

## Case history

An 84-year-old woman presented to our hospital with a history of sudden onset upper abdominal pain, vomiting and abdominal distension. Prior to this, she had not opened her bowels or passed flatus for two days. She was a smoker of several years and had been treated for an exacerbation of chronic obstructive pulmonary disease with steroids, antibiotics and bronchodilators. Apart from hypertension and arthritis, there was no other medical or surgical history. On examination, she was acutely unwell with tachypnoea (28 respirations per minute) and mild tachycardia (98 beats per minute) but was normotensive and apyrexial. Her abdomen was distended with features of generalised peritonitis but there was no palpable mass or abdominal aortic aneurysm. Rectal examination was unremarkable. Blood tests revealed a compensated metabolic acidosis, elevated lactate at 2.5mmol/l, leucocytosis of 23,100 × 10^6^/l, urea of 19.6mmol/l and creatinine of 154μmol/l.

**Figure 1:**
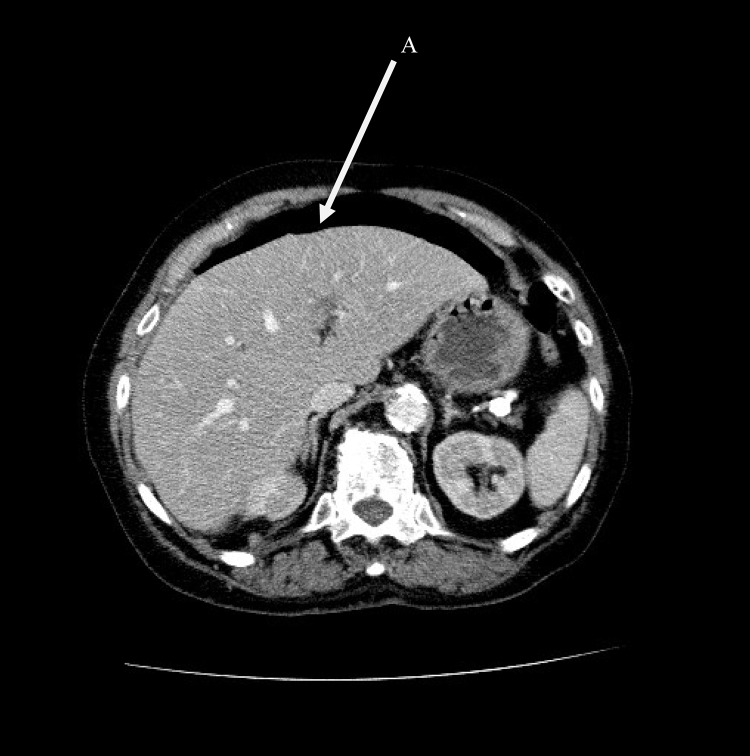
Axial computed tomography showing free intraperitoneal air (A) in the upper abdomen, suggestive of gastrointestinal perforation

Urgent computed tomography (CT) reported the presence of free intraperitoneal air mostly in the upper abdomen and some free fluid in the pelvis. Diverticular disease was noted throughout the sigmoid colon as well as a large cystic mass in the posterior part of the uterus. It was not possible to confirm the specific site of perforation accurately but, in the context of the presenting history, it was suggestive of either a perforated peptic ulcer or diverticulitis (Fig 1).

Following prompt resuscitation and intravenous antibiotics, a laparotomy was performed, which confirmed the presence of free air and pus as well as an inflamed sigmoid colon and dilated large bowel. Perforated diverticulitis was henceforth suspected but thorough laparotomy revealed no gastrointestinal (GI) perforation. However, closer inspection of the pelvic organs by both the colorectal and gynaecology teams confirmed the aetiology as pyometra with perforation of the anterior wall of the uterus. The posterior wall of the uterus also contained a large firm mass partially adherent to the rectum.

**Figure 2:**
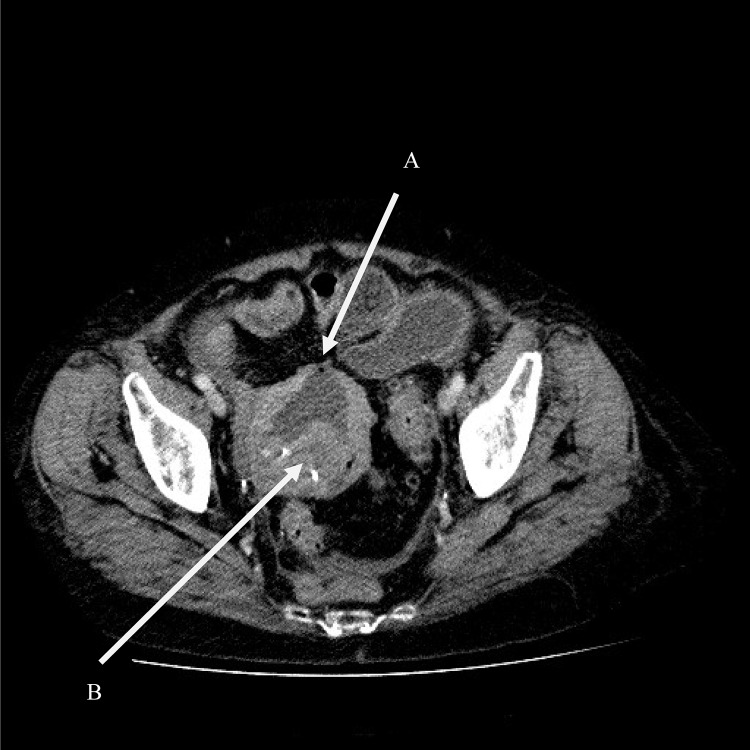
Axial computed tomography showing a fluid filled uterus with a perforation and free air in the anterior wall (A). Leiomyomata can also be seen on the posterior wall (B).

**Figure 3:**
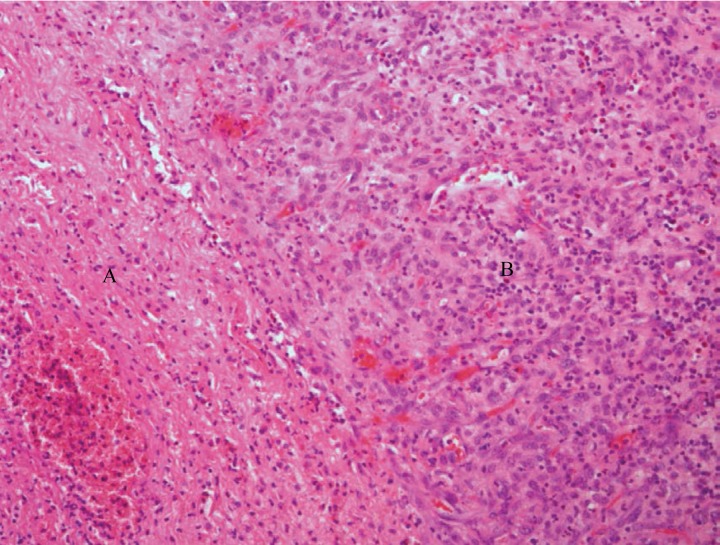
Histological examination demonstrating endometrial inflammatory exudates (A) and granulation tissue (B). Haematoxylin and eosin stain (100x magnification).

**Figure 4:**
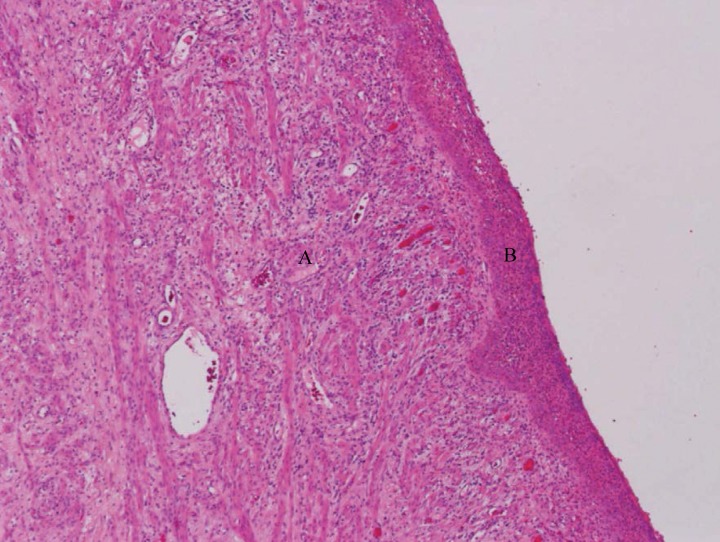
Histological examination demonstrating inflamed myometrium (A) and peritonitis (B). Haematoxylin and eosin stain (40x magnification).

A total abdominal hysterectomy, copious saline lavage and tube drainage was performed as a joint procedure. No colonic resection was deemed necessary. Retrospective review of the CT at the colorectal multidisciplinary team meeting reported, in addition to the previously documented findings, a fluid filled uterus with a perforation and free air in the anterior wall as well as a lesion in the posterior wall in keeping with fibroids (Fig 2). Histological examination confirmed pyometra and multiple partly hyalinised leiomyomas but no evidence of malignancy (Figs 3 and 4).

Post-operatively, intravenous antibiotics were continued and the patient was supported in the intensive care unit for a short period prior to stepping down to ward care and making a full recovery. Her home and social circumstances contributed to a prolonged period of convalescence and she was discharged home after 35 days.

## Discussion

Pyometra, an accumulation of pus in the uterine cavity, has an estimated incidence of 0.01–0.5% and occurs due to inadequate drainage of endometrial secretions through the cervix.^1^ Malignancy and the sequelae of radiotherapy is the most common cause. It occurs predominantly in elderly post-menopausal women where the incidence may be as high as 13.6%. Benign and congenital conditions of the cervix as well as intrauterine devices (IUDs) are also known to cause pyometra. The classic presentation is with the triad of purulent vaginal discharge, post-menopausal bleeding and lower abdominal pain.

Spontaneous perforation of the uterus is also rare and has two peaks of its incidence: first, in women of child bearing age and associated with a gravid uterus or presence of an IUD; second, in elderly post-menopausal women and associated with pyometra.^2^ Spontaneously perforated pyometra is rare with only 36 documented cases up to 2011, many of which occurred in Eastern Asia. Malignancy was the cause in 11 cases (30%), leiomyoma in 2 (5%) and there was no apparent cause in the remaining cases.^2^

Abdominal pain, vomiting and fever predominate as the presenting symptoms in spontaneously perforated pyometra while gynaecological symptoms such as vaginal bleeding or discharge occur in less than 10%.^2^ Generalised peritonitis (47.4%) and GI perforation (36.8%) are the most prevalent pre-operative diagnoses with radiological findings of pneumoperitoneum present in only half of these. The presence of pneumoperitoneum secondary to spontaneously perforated pyometra is an interesting yet confusing finding given the absence of GI perforation. GI perforation is the cause of pneumoperitoneum in 85–95% of cases and requires surgical intervention as its definitive management.^3,4^

Other ‘non-surgical’ causes of pneumoperitoneum are by and large iatrogenic and can be managed conservatively. Free intraperitoneal air is seen commonly following open or laparoscopic surgery, peritoneal dialysis and its complications, percutaneous endoscopic gastrostomy tube placement,and spontaneous bacterial peritonitis.^5^ Pneumoperitoneum that is not associated with GI perforation or of iatrogenic origin is, however, rare.

In our case there are two possible explanations for the pneumoperitoneum. First, the presence of free intraperitoneal air may be due to gas forming organisms. Previous reviews report that *Escherichia coli* and *Bacteroides fragilis* are the most commonly associated organisms.^2^ However, in this case no specific bacterial culture was obtained at laparotomy. Whether the presence of frank pus and gas-forming organisms in the peritoneal cavity may have contributed such a significant volume of free air is of further interest. Second, the passage of air through the genital canal into the peritoneal cavity is well documented.^5^ Nevertheless, the pathophysiology of spontaneously perforated pyometra is reliant on a closed or stenosed cervix and, together with the absence of any gynaecological symptoms, the passage of transcervical air is unlikely in this case.

Pre-operative diagnosis of spontaneously perforated pyometra is difficult but the initial management remains the same as for generalised peritonitis or GI perforation: prompt resuscitation, antibiotics and radiological investigation. While CT is often indicated in such circumstances, this case demonstrates that accurate radiological diagnosis of spontaneously perforated pyometra can still be difficult, especially when imaging is reviewed in the acute setting and often not by a specialist abdominopelvic radiologist. Given the absence of gynaecological symptoms in most cases, diagnosis is frequently not possible until laparotomy is performed.

Total abdominal hysterectomy, with or without bilateral salpingo-oophorectomy, and copious peritoneal lavage is the definitive surgical treatment. Colonic resection may also be necessary in cases of colouterine fistulation or invasive malignancy. Mortality from spontaneously perforated pyometra exceeds 40% and, again, highlights the importance of multidisciplinary involvement in treating sepsis.^2^

## Conclusions

This case illustrates several lessons for the general surgeon, who is likely to be referred patients with pneumoperitoneum. Although an uncommon finding, spontaneously perforated pyometra should feature as part of the differential diagnoses for elderly post-menopausal women presenting with an acute abdomen and pneumoperitoneum. An appreciation of acute and neoplastic gynaecological disease is essential as many patients with gynaecological pathologies may present with signs and symptoms mimicking those of GI origin. Most patients with spontaneously perforated pyometra will require surgery and so a good appreciation of the pelvic anatomy and close collaboration with gynaecology colleagues is essential. Meanwhile, meticulous clinical judgement is required in discerning which patients may benefit from conservative management.
